# The Role of Human Papillomaviruses and Polyomaviruses in BRAF-Inhibitor Induced Cutaneous Squamous Cell Carcinoma and Benign Squamoproliferative Lesions

**DOI:** 10.3389/fmicb.2018.01806

**Published:** 2018-08-14

**Authors:** Karin J. Purdie, Charlotte M. Proby, Hasan Rizvi, Heather Griffin, John Doorbar, Mary Sommerlad, Mariet C. Feltkamp, Els Van der Meijden, Gareth J. Inman, Andrew P. South, Irene M. Leigh, Catherine A. Harwood

**Affiliations:** ^1^Centre for Cell Biology and Cutaneous Research, Blizard Institute, Barts and the London School of Medicine and Dentistry, Queen Mary University of London, London, United Kingdom; ^2^Division of Cancer Research, School of Medicine, University of Dundee, Dundee, United Kingdom; ^3^Department of Pathology, Barts Health NHS Trust, London, United Kingdom; ^4^Division of Virology, Department of Pathology, University of Cambridge, Cambridge, United Kingdom; ^5^Department of Dermatology, Barts Health NHS Trust, London, United Kingdom; ^6^Department of Medical Microbiology, Leiden University Medical Center, Leiden, Netherlands; ^7^Department of Dermatology and Cutaneous Biology, Thomas Jefferson University, Philadelphia, PA, United States; ^8^Institute of Dentistry, Barts and the London School of Medicine and Dentistry, Queen Mary University of London, London, United Kingdom

**Keywords:** human polyomaviruses, human papillomaviruses, cutaneous squamous cell carcinomas, BRAF inhibitors, melanoma

## Abstract

**Background:** Human papillomavirus (HPV) has long been proposed as a cofactor in the pathogenesis of cutaneous squamous cell carcinoma (cSCC). More recently, the striking clinico-pathological features of cSCCs that complicate treatment of metastatic melanoma with inhibitors targeting BRAF mutations (BRAFi) has prompted speculation concerning a pathogenic role for oncogenic viruses. Here, we investigate HPV and human polyomaviruses (HPyV) and correlate with clinical, histologic, and genetic features in BRAFi-associated cSCC.

**Materials and Methods:** Patients receiving BRAFi treatment were recruited at Barts Health NHS Trust. HPV DNA was detected in microdissected frozen samples using reverse line probe technology and degenerate and nested PCR. HPV immunohistochemistry was performed in a subset of samples. Quantitative PCR was performed to determine the presence and viral load of HPyVs with affinity for the skin (HPyV6, HPyV7, HPyV9, MCPyV, and TSPyV). These data were correlated with previous genetic mutational analysis of H, K and NRAS, NOTCH1/2, TP53, CDKN2A, CARD11, CREBBP, TGFBR1/2. Chromosomal aberrations were profiled using single nucleotide polymorphism (SNP) arrays.

**Results:** Forty-five skin lesions from seven patients treated with single agent vemurafenib in 2012–2013 were analyzed: 12 cSCC, 19 viral warts (VW), 2 actinic keratosis (AK), 5 verrucous keratosis/other squamoproliferative (VK/SP) lesions, one melanocytic lesion and 6 normal skin samples. Significant histologic features of viral infection were seen in 10/12 (83%) cSCC. HPV DNA was detected in 18/19 (95%) VW/SP, 9/12 (75%) cSCC, 4/5 (80%) SP, and 3/6 (50%) normal skin samples and in 1/12 cases assessed by immunohistochemistry. HPyV was co-detected in 22/30 (73%) of samples, usually at low viral load, with MCPyV and HPyV7 the most common. SNP arrays confirmed low levels of chromosomal abnormality and there was no significant correlation between HPV or HPyV detection and individual gene mutations or overall mutational burden.

**Conclusion:** Despite supportive clinicopathologic evidence, the role for HPV and HPyV infection in the pathogenesis of BRAFi-induced squamoproliferative lesions remains uncertain. Synergistic oncogenic mechanisms are plausible although speculative. Nonetheless, with the prospect of a significant increase in the adjuvant use of these drugs, further research is justified and may provide insight into the pathogenesis of other BRAFi-associated malignancies.

## Introduction

Human papillomaviruses (HPV), particularly those of the beta genus (beta-PV), have long been proposed as cofactors with ultraviolet radiation in the pathogenesis of cutaneous squamous cell carcinoma (cSCC), especially those associated with the rare genodermatosis, epidermodysplasia verruciformis and with immune suppression (Wang et al., 2014; Howley and Pfister, 2015; Quint et al., 2015; Harwood et al., 2017). Over the past decade, 13 human polyomaviruses (HPyV) have been identified and classified (Calvignac-Spencer et al., 2016) and those with affinity for the skin include HPyV6, HPyV7, HPyV9, the trichodysplasia spinulosa-associated polyomavirus (TSPyV), the Merkel cell polyomavirus (MCPyV) (DeCaprio and Garcea, 2013; Feltkamp et al., 2013; van der Meijden et al., 2010, 2013; Nguyen et al., 2017) and the Lyon IARC polyomavirus (LIPyV) (Gheit et al., 2017). MCPyV was the first to be associated with malignancy – the aggressive cutaneous neuroendocrine cancer Merkel cell carcinoma (Feng et al., 2008; Shuda et al., 2008) – and has also been investigated in the context of cSCC (Dworkin et al., 2009; Scola et al., 2012). More recently, the clinical and histologic features of cSCCs and other squamoproliferative lesions that complicate treatment for metastatic melanoma with oral small molecule *BRAF* inhibitors (BRAFi) have raised the possibility of significant viral involvement in their pathogenesis (Boussemart et al., 2013). Both HPV and HPyV have been investigated, but studies have to date provided conflicting evidence for their role. With the prospect of future widespread adjuvant use of these drugs (Long et al., 2017; Maio et al., 2018), a more detailed understanding of the pathogenesis of BRAFi-induced cSCC (BRAFi-SCC) remains important.

Oncogenic mutations in the oncoprotein *BRAF*, which encodes the growth signal transduction serine/threonine protein kinase B-Raf, are found in approximately 50% of melanomas and result in constitutive activation of the RAS/mitogen-activated protein kinase (MAPK) pathway. The most common BRAF mutation results in a substitution of a valine (V) residue to glutamic acid (E) at amino acid position 600 (V600E) (Long et al., 2011). This locks the kinase into the active conformation and results in melanocyte hyperproliferation. BRAF inhibitors have been developed that exploit this mutation and competitively bind to the active conformation of the kinase (Zhang et al., 2009; Ribas and Flaherty, 2011) Vemurafenib and dabrafenib are two such selective small molecule inhibitors of oncogenic *BRAF* and are associated with high response rates and improved progression-free survival and overall survival compared with chemotherapy in patients with BRAFV600 mutated melanoma (Chapman et al., 2017). However, responses are generally temporary, with a median time to relapse of approximately 6 months. Vemurafenib entered routine clinical use in 2011/2012 and has been associated with various cutaneous adverse effects. These include rashes, photosensitivity, hyperkeratosis and development of *de novo* squamoproliferative lesions in 16–26.7% of patients, ranging from benign VW and squamous papillomas/verrucous keratoses (VK) to keratoacanthomas (KA) and cSCC (Flaherty et al., 2010; Sosman et al., 2012; Anforth et al., 2013; Blank et al., 2017; Chapman et al., 2017). The median time to presentation for BRAFi-SCC is 8–12 weeks and it is argued that this rapid timeframe points to pre-existing mutations being given a selective advantage due to BRAFi treatment, rather than mutations arising *de novo* due to therapy. Consistent with this mechanism, there is evidence for paradoxical hyperactivation of the MAPK pathway in cells with wild-type BRAF but mutated RAS through allosteric and catalytic mechanisms that relieve the auto-inhibition of wild-type RAF kinase (Hatzivassiliou et al., 2010; Heidorn et al., 2010; Poulikakos et al., 2010). Indeed, many of these BRAFi features overlap with the cutaneous manifestations of RASopathies – genetic diseases such as cardiofaciocutaneous and Costello syndromes characterized by activating germ line mutations in RAS (Rinderknecht et al., 2013; Sfecci et al., 2017). Also consistent with this, 18–60% of BRAFi-cSCC have somatic mutations in HRAS or KRAS, which is significantly higher than in sporadic cSCC (Oberholzer et al., 2012; Su et al., 2012; South et al., 2014). Nevertheless, many BRAFi-cSCC are RAS wild type, and RAS mutations have been also been detected in benign epithelial skin lesions (South et al., 2014; Hassel et al., 2015), suggesting that accelerated oncogenesis of RAS-mutated cells is not the only aetiologic mechanism and that additional cofactors may be involved. Attention has focused on infectious agents, particularly oncogenic viruses. HPV has been the main candidate given its previous proposed role in EV and immune suppression-associated cSCCs, coupled with evidence in BRAFi-cSCC of clinical and histological patterns of viral wart-like features and overexpression of p16 (Boussemart et al., 2013). Human polyomaviruses, particularly MCPyV, have also been investigated but, to date, the available evidence for both HPV and HPyV remains inconclusive (Anforth et al., 2012; Chu et al., 2012; Boussemart et al., 2013; Ganzenmueller et al., 2013; Falchook et al., 2013, 2016; Ko et al., 2013; Frouin et al., 2014; Holderfield et al., 2014; Schrama et al., 2014; Cohen et al., 2015; Dika et al., 2015; Viarisio et al., 2017a,b).

Single agent BRAFi therapy has generally been replaced by combination BRAFi and MEK inhibition (MEKi): phase III studies demonstrated improved clinical outcomes and significantly delayed resistance compared with BRAFi alone and BRAFi-MEKi combination therapy (vemurafenib/cobimetanib and dabrafenib/trametinib) is now the standard of care for BRAF mutated metastatic melanoma (Flaherty et al., 2012; Larkin et al., 2014). Combination therapy also results in decreased incidence of BRAFi-SCC to around 4%, which may be due to the fact that MEKi bypasses the point of paradoxical RAF activation (Dummer et al., 2012). Although rates of BRAFi-SCC are consequently reduced with combination therapy, these drugs are now being introduced as adjuvant treatment in high-risk, non-metastatic primary melanoma (stages IIC–IIIA–IIIB–IIIC). In the recent BRIM-8 study of adjuvant vemurafenib in BRAF-mutated melanoma, 16% of patients treated with adjuvant vemurafenib had BRAFi-cSCC or KA (Maio et al., 2018). This incidence is considerably lower with BRAFi-MEKi combination adjuvant therapy (Long et al., 2017), but with approval for adjuvant treatment comes the prospect of a huge increase in the numbers of patients receiving these agents in the near future. The need to better understand the pathogenesis of their associated cSCC therefore remains important.

In this study we have examined HPV and HPyV status in a series of benign and malignant BRAFi-associated skin lesions and correlated these data with key clinical, histologic and genetic parameters in order to further investigate the contribution of these viruses to the pathogenesis of BRAFi-induced skin tumors.

## Materials and Methods

### Patients and Samples

Patients were recruited from the melanoma clinic at Barts Health NHS Trust. Punch biopsies were taken after surgical excision of lesions or from bisected shave biopsies. They were immediately snap-frozen in liquid nitrogen and stored at −80°C. The remainder of the tissue was sent for formalin fixation and histologic diagnosis. In order to enrich for tumor cell populations, fresh-frozen samples were laser-capture microdissected using the Zeiss Palm Microbeam microscope (Zeiss, Cambridge, United Kingdom). Depending on sample size and purity, as estimated from a reference hematoxylin and eosin slide, between 30 and 60 sections of 8 mm thickness were cut onto 1.0 mm PEN membrane slides (Zeiss), stained in 0.05% acid fuchsin (Acros Organics, Morris Plains, NJ, United States) in distilled water and 0.05% toluidine blue O (Acros Organics, Morris Plains, NJ, United States) in 70% ethanol, and microdissected, with tumor cells collected into 180 μl ATL buffer (Qiagen, Crawley, United Kingdom). All sections were cut using a fresh microtome to prevent cross-contamination. DNA extraction was performed using the QIAamp DNA micro kit (Qiagen, Crawley, United Kingdom) according to the manufacturer’s instructions. To provide a source of germline DNA, paired venous blood samples were obtained concomitantly with lesional tissue and stored at −80°C before DNA extraction using the QIAamp DNA blood mini kit (Qiagen, Crawley, United Kingdom). The quality of the extracted DNA was assessed by β-globin reference gene PCR.

### Histopathology

Histology sections were prepared from formalin-fixed paraffin-embedded tissue and stained with hematoxylin and eosin under standard conditions. All diagnoses were confirmed after review by an experienced dermatopathologist (HR). Samples were scored as having features consistent with viral infection if koilocytosis was observed in conjunction with at least three of the following five features: acanthosis, hypergranulosis, parakeratosis, hyperkeratosis, and typical papillomatous architecture. Consensus scoring of viral features was reached with two additional observers (KP, CH).

### HPV Detection and Genotyping

Beta, gamma, alpha, mu, nu, and novel HPV types were detected using a comprehensive panel of HPV detection and typing methodologies. The presence of beta-HPV was investigated using RHA kit skin (beta) HPV detection system (de Koning et al., 2006) according to the manufacturer’s instructions (Diassay, Rijswijk, The Netherlands). In addition, the RHA kit HPV SPF10-LiPA25 (Labo Bio-medical products BV, Rijswijk, The Netherlands) was used to detect the presence of 25 high- and low-risk mucosotropic alpha-HPVs, according to the manufacturer’s instructions. Degenerate nested PCR protocols were used to investigate the presence of cutaneous alpha-HPV and mu and nu genera (Harwood et al., 1999) and the gamma genus (Forslund et al., 1999; Antonsson et al., 2000).

### Polyomavirus Detection and Genotyping

In lesional samples for which sufficient DNA was available, quantitative PCR was performed as previously described (van der Meijden et al., 2014, 2017), to determine the presence and load of HPyV9, MCPyV, and TSPyV. For HPyV6 and HPyV7 quantitative PCR the following primers and probes located in VP1 were used: 5′-GTAGGGTATGCTGGTAAC-3′ (HPyV6 sense), 5′-CAGGAATTGTCTAAACATCATATC-3′ (HPyV6 antisense), 5′-CTCTCCTCTGTCTGAAGTGAACTC-TAA-3′ (HPyV6 probe), 5′-GTGCTGATATGGTTGGAA-3′ (HPyV7 sense), 5′-TCTGCAGTGGACTCTAAA-3′ (HPyV7 antisense), 5′-AGCCTGTACTGTTCTCTGGTTACT-3′ (HPyV7 probe). Input cell equivalents were determined by normalization to beta-globin.

All DNA extractions and PCRs for both HPV and HPyV were performed using standard operating procedures designed to reduce the possibility of contamination (Harwood et al., 1999). DNA extraction, water and buffer PCR controls were used to exclude contamination and these were consistently negative.

### Immunohistochemistry

Immunofluorescence analysis was carried out using polyclonal antibodies raised against the E4 proteins of HPV 5, 8, and 23 (beta-PV types), or the E4 protein of HPV2 and 57 (alpha-PV types) using the protocols previously described (Griffin and Doorbar, 2016). In the double staining experiments, a monoclonal antibody (8H3) prepared against the HPV 8 E4 protein was used in place of the beta-PV E4 polyclonal antibodies. Polyclonal and monoclonal antibodies were prepared against GST-E4 fusion proteins (Doorbar et al., 1997; Borgogna et al., 2012). Techniques for the overlay of fluorescence staining patterns onto the hematoxylin and eosin sections have been described previously (Griffin et al., 2015). All tissue sections were formalin fixed prior to staining. Sections were counterstained with DAPI to visualize cell nuclei.

### SNP Array Analysis of Gross Chromosomal Aberrations

Cutaneous squamous cell carcinoma and paired venous blood DNA samples were subjected to the GeneChip Genome-Wide Human SNP Array 6.0 assay (Affymetrix Inc., Santa Clara, CA, United States) according to the manufacturer’s protocol. Processing was performed as previously described (Teh et al., 2005) using the Genome Oriented Laboratory File (GOLF) system for the analysis and display of single nucleotide polymorphism (SNP) signal data. Copy number profiles of vemurafenib-associated cSCC were compared with those observed in a previous study of sporadic cSCC (Purdie et al., 2009).

### Genetic Mutational Analysis

Targeted genetic analysis of all samples was undertaken using 454 pyrosequencing performed using the GS Junior system (Roche/454 Life Sciences, Branford, CT, United States) and Fluidigm (Fluidigm Corporation, San Francisco, CA, United States) PCR amplicon libraries as template. In addition to *H, K*, and *NRAS*, we also analyzed the genes *NOTCH1, NOTCH2, TP53, CDKN2A, CARD11, CREBBP, TGFBR1*, and *2*, all of which our previous research has implicated in the pathogenesis of cSCC (Brown et al., 2004; South et al., 2014; Cammareri et al., 2016; Watt et al., 2015, 2016). Primers were designed and validated by Fluidigm (Fluidigm Corporation, San Francisco, CA, United States) as per recommended guidelines for Roche Titanium sequencing (Roche, Mannheim, Germany). Variant detection required a minimum of four supporting reads and a minimum variant allele frequency threshold of 0.1. Detailed genetic analysis of these samples has previously been reported (South et al., 2014; Cammareri et al., 2016).

### Ethical Approval

This study was carried out in accordance with the recommendations of East London and City Health Authority local ethics committee. The protocol was approved by the East London and City Health Authority local ethics committee. All subjects gave written informed consent in accordance with the Declaration of Helsinki.

## Results

### Patients

**FIGURE 1:**
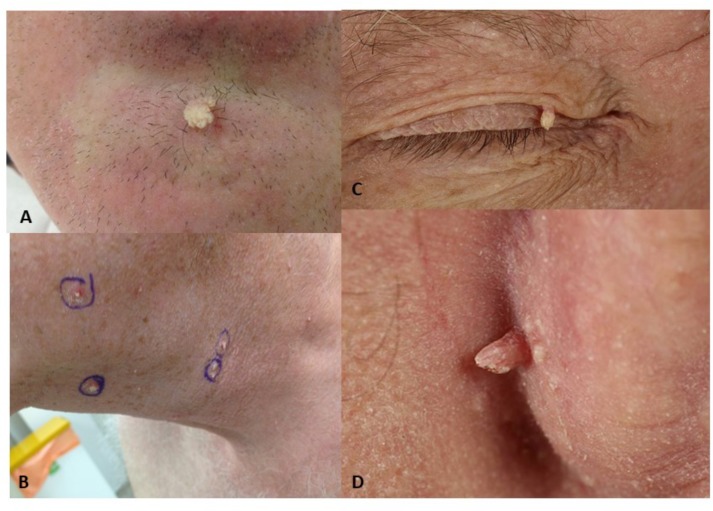
Viral-wart like lesions developing after BRAFi exposure. **(A)** Lesion V10, viral wart on chin of patient 2; **(B)** Viral warts on the neck and chin of patient 1; **(C)** Viral wart on the eyelid of patient 5; **(D)** Lesion V22, post-auricular viral wart in patient 3.

**FIGURE 2:**
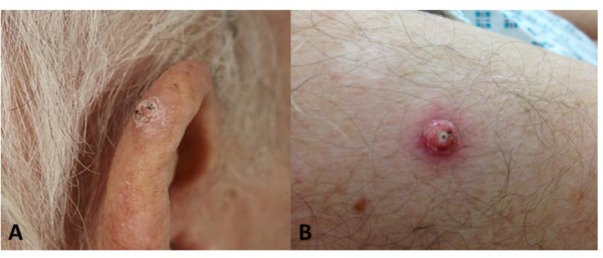
Cutaneous squamous cell carcinomas developing after BRAFi exposure. **(A)** Well-differentiated SCC on the ear of patient 1; **(B)** Lesion V4, well-differentiated SCC on the arm of patient 1.

A total of seven patients with 45 skin lesions were recruited (**Figures 1**, **2** and **Table 1**). They included 4 men (mean age 61.25 years, range 35–87 years) and 3 women (mean age 56.3 years, range 33–82 years). All patients had metastatic melanoma with V600E BRAF mutations. All were treated in 2012–2013 with single agent vemurafenib. For all patients, samples were collected at first presentation with skin lesions after starting vemurafenib. The mean time to either disease progression (*n* = 3 patients) or death from melanoma whilst receiving vemurafenib (*n* = 4 patients) was 7.7 months (range 3–17 months).

**Table 1:** Clinical, histologic, viral, and genetic features of the seven patients and 45 lesions analyzed.

Pt	Sex/age (years)	Lesion code	Weeks on BRAFi^1^ (weeks)	Body site	SE/NSE^2^	Diagnosis^3^	Viral features^4^	Mutations^5^	Beta-PV^6^	Alpha-PV	Gamma-PV	IHC^7^	HPyV6^8^	HPyV7^8^	HPyV9^8^	TSPyV^8^	MCPyV^8^
**1**	M/67	V1	9	Scalp	SE	VW	Y	NOTCH1, CREBBP	8, 9, 36, 38, 111	28		ND	7.5 × 10^−6^	3.6 × 10^−2^	Neg	Neg	1.7 × 10^−2^
		V2	9	Suprapubic	NSE	V K	Y	NOTCH1, HRAS, CARD11	8, 24, 36, 92, 111			ND	ND	ND	ND	ND	ND
		V3	9	Post-auricular	SE	VW	Y	TGFBR2, CDKN2a, CARD11, NOTCH2, CREBBP	8, 36			ND	ND	ND	ND	ND	ND
		V4	9	Arm	SE	SCC	Y	TGFBR1, NOTCH1, HRAS	8, 12, 24, 36, 111, FA51			ND	Neg	1.7 × 10^−4^	Neg	Neg	4.6 × 10^−2^
		V5	9	Inner Thigh	NSE	VW	Y	NOTCH1, NOTCH2, HRAS, CREBBP	8, 12, 36, 111			ND	2.9 × 10^−4^	Neg	Neg	Neg	2.8 × 10^−2^
		V6	9	Lower Back	NSE	SCC	Y	TP53, HRAS	12, 36, 92			ND	5.12 × 10^−5^	1.3 × 10^−1^	Neg	Neg	3.9 × 10^−3^
		V7	9	Shoulder	NSE	SCC	Y	NOTCH1, TP53, TGFBR1, CREBBP	15, 24, 36, 76, 92, 107, FA84			Neg	Neg	3.9 × 10^−5^	Neg	Neg	1.8 × 10^−2^
		V8	9	Posterior neck	SE	VK	Y	NOTCH1, CARD11, TGFBR1	8, 24, 36, 93			Neg	4.6 × 10^−5^	4.8 × 10^−4^	Neg	Neg	2.3 × 10^−1^
		V9	9	Cheek	SE	VW	Y	HRAS, TP53^∗^, NOTCH1,^∗^ NOTCH2^∗^	8, 12, 24, 36, 76, 92 93	28		ND	ND	ND	ND	ND	ND
		V11	13	Scalp	SE	VW	Y	NOTCH1, TGFBR2, CARD11, CREBBP	8,23, 36, 92, 93			Neg	Neg	2.2 × 10^−4^	Neg	Neg	1 × 10^−1^
		V12	13	Abdomen	NSE	N-NSE	N	NOTCH1	5, 92			ND	ND	ND	ND	ND	ND
		V13	13	Lower back	NSE	SCC	Y	Nil	8, 24, 92, 107, 111, FA14			Neg	3.9 × 10^−5^	2.4 × 10^−4^	Neg	Neg	5.9 × 10^−3^
		V14	13	Popliteal Fossa	NSE	VW	Y	NOTCH1, CREBBP	12, 24, 36			ND	Neg	1.3 × 10^−2^	Neg	Neg	1.7 × 10^−3^
		V15	13	Post-auricular	SE	VW	Y	CARD11	93			ND	Neg	1.6 × 10^−3^	Neg	Neg	5.6 × 10^−2^
		V16	13	Abdomen	NSE	VW	Y	TP53, HRAS	8, 36, 92, 93			ND	Neg	6, × 10^−4^	Neg	Neg	9.9 × 10^−3^
		V17	13	Abdomen	NSE	VW	Y	NOTCH1, CDKN2a	8, 36, 92, 113			ND	Neg	1.4 × 10^−1^	Neg	Neg	6.2 × 10^−4^
		V18	14	Chest	NSE	VW	Y	NOTCH1, HRAS, KRAS	8^∗∗^, 12, 24, 76, 92^∗∗^, 111			Neg	1.12 × 10^−5^	5, × 10^−5^	Neg	Neg	5.8 × 10^−3^
		V19	14	Groin	NSE	SCC	Y	HRAS, NOTCH1	8, 12, 24, 36, 92			Neg	3.93 × 10^−6^	1.4 × 10^−4^	Neg	Neg	3.6 × 10^−3^
		V20	14	Chest	SE	SCC	Y	Nil	8, 12, 24, 92^∗∗^, 93			Neg	1.29 × 10^−5^	2.4 × 10^−4^	Neg	Neg	1.2 × 10^−2^
		V25	18	Arm	SE	N-P V27	N	NOTCH1	Neg			ND	ND	ND	ND	ND	ND
		V26	18	Abdomen	NSE	VW	Y	CARD11, NOTCH1, HRAS, CREBBP	8, 92			Neg	Neg	5.9 × 10^−5^	Neg	Neg	5.3 × 10^−3^
		V27	18	Upper arm	SE	SCC	Y	HRAS, CARD11, CREBBP, NOTCH1 NOTCH2^∗^ KRAS^∗^ TP53^∗^	Novel			Neg	Neg	Neg	Neg	Neg	Neg
		V28	18	Lower abdomen	NSE	SCC	Y	NOTCH2, NOTCH1^∗^	Neg			Neg	ND	ND	ND	ND	ND
		V31	34	Cheek	SE	VK	N	Nil	Novel			ND	3.66 × 10^−6^	2.4 × 10^−4^	Neg	Neg	7.6 × 10^−3^
		V32	34	Upper Arm	SE	VW	Y	NOTCH1, NOTCH2, HRAS, CREBBP, TP53^∗^	24^∗∗^, 36, 92, 93			ND	ND	ND	ND	ND	ND
**2**	M/35	V10	13	Chin	SE	VW	Y	Nil	8, 12, 80^∗∗^, 76	57^∗∗^		57, beta	ND	ND	ND	ND	ND
		V29	28	Scapula	NSE	VW	Y	NOTCH2, CREBBP	12^∗∗^, 8, 20, 23, 36,76, 80, 92, 96	57		ND	ND	D	ND	ND	ND
**3**	M/56	V21	8	Neck	SE	VK	N	HRAS, CARD11, NOTCH1 ^∗^ NOTCH2^∗^	22, 115, 150			ND	3.73 × 10^−5^	6.4 × 10^−5^	Neg	Neg	Neg
		V22	14	Post-auricular	SE	VW	Y	TGFBR1, HRAS NOTCH2^∗^	22, 107			ND	ND	ND	ND	ND	ND
		V23	14	Upper arm	SE	SCC	Y	CREBBP, CARD11, TGFBR1, NOTCH2^∗^	2, 14D, 22, 38, 107, 115			Neg	Neg	Neg	Neg	Neg	1.3 × 10^−4^
		V24	14	Upper Arm	SE	N-P to V23	N	NOTCH1	Novel			ND	ND	ND	ND	ND	ND
**4**	F/33	V30	16	Abdomen	NSE	SCC	N	TP53, HRAS, NOTCH1^∗^ NOTCH2^∗^	Neg		FA49	ND	Neg	Neg	Neg	Neg	Neg
**5**	M/87	V33	6	Ear	SE	VW	Y	NOTCH1, TP53	9			ND	Neg	Neg	Neg	Neg	Neg
		V34	6	Chin	SE	AK	Y	Nil	9, 38			ND	Neg	1.5 × 10^−5^	Neg	Neg	Neg
**6**	F/82	V35	12	Neck	SE	SCC	Y	TGFBR1, TGFBR2, NOTCH1, TP53, KRAS, NOTCH2, CARD11, CREBBP	Neg			ND	Neg	Neg	Neg	Neg	Neg
		V36	12	Neck	SE	N-P V35	N	Nil	Neg			ND	ND	ND	ND	ND	ND
		V37	12	Neck	SE	N -SE	N	NOTCH1, CARD11	Neg			ND	ND	ND	ND	ND	ND
		V38	12	Shoulder	NSE	VW	Y	TGFBR1, TGFBR2, TP53, KRAS, NRAS, CARD11, CREBBP	Neg			ND	ND	ND	ND	ND	ND
		V39	12	Lower Leg	SE	SP	Y	TGFBR1, NOTCH1, NOTCH2,, TP53, CREBBP	Neg			ND	Neg	Neg	Neg	Neg	1.5 × 10^−3^
		V40	12	Arm	SE	SCC	Y	TGFBR2, CREBBP	Neg			ND	Neg	Neg	4 × 10^−4^	Neg	2.9 × 10^−3^
**7**	F/54	V41	11.5	Neck	SE	N-SE	N	NOTCH2, CREBBP	Novel			ND	ND	ND	ND	ND	ND
		V42	11.5	Back	NSE	VW	Y	TP53	80			ND	Neg	Neg	Neg	Neg	Neg
		V43	11.5	Back	NSE	M		NOTCH1, NOTCH2, NRAS, CARD11, CREBBP	80			ND	Neg	Neg	Neg	Neg	9 × 10^−4^
		V44	11.5	Back	NSE	VW	Y	CARD11	80^∗∗^			ND	Neg	Neg	Neg	Neg	Neg
		V45	11.5	Back	NSE	AK	Y	NOTCH1, TP53	80			ND	Neg	Neg	Neg	Neg	1.4 × 10^−4^

*^1^Weeks on BRAFi: number of weeks on vemurafenib at the time lesion was removed. ^2^SE/NSE: Sun exposed or non-sun exposed body site. ^3^Diagnosis: SCC – well differentiated SCC; V, viral wart; VK, verrucous keratosis; AK, actinic keratosis; N-P, normal skin perilesional to; N-SE, normal skin sun exposed; N-NSE – normal skin non sun exposed; M, benign melanocytic; SP, squamoproliferative lesion with viral features and dysplasia but no clear evidence of invasion. ^4^Viral features: Y, yes; N, no: yes if koilocytosis was observed in conjunction with at least 3 of the following 5 features: acanthosis, hypergranulosis, parakeratosis, hyperkeratosis and typical papillomatous architecture. ^5^Mutations: these have previously been published (South, 2014; Cammareri, 2016). ^∗^ indicates lesion bisected and results represent total mutations found across both portions. ^6^Beta-PV: beta HPV types. Beta-PV types associated with strong bands on the RH assay are recorded as ^∗∗^; neg, negative. ^7^ IHC; immunohistochemistry – performed on 12 selected lesions. ^8^HPyV6, -7, -9, TSPyV and MCPyV: neg, negative; N/D, not done; values recorded are viral copies per cell.*

### Samples (**Table 1**)

Samples consisted of 12 SCC, 19 VW, 2 actinic keratoses (AK), one melanocytic lesion and 6 non-lesional skin samples (3 normal skin and 3 normal skin perilesional to cSCC), 4 verrucous keratoses (VK – squamous papillomas, some with viral features but without evidence of dysplasia) and one squamoproliferative (SP) lesion with viral features and dysplasia but no clear invasion (SP). Lesions were from both chronically sun-exposed (SE) and non-chronically sun-exposed (NSE) sites. SE sites included head and neck, arms, and lower legs in females. NSE included all other sun-protected sites. Lesions from SE sites included 6/12 (50%) cSCCs, 5/5 VKs, 9/19 (47%) VWs, and 1/2 AKs. The normal skin samples comprised 2 SE, one NSE and 3 perilesional to SCCs on SE sites.

The mean time to development of biopsy-proven lesions after initiation of vemurafenib was 8.75 weeks for AK (range 6–11.25), 9.7 weeks for VK/SP (range 8–12), 9.9 weeks for VW (range 6–13), and 11.25 weeks for cSCC (range 8–16 weeks). For 2 patients, additional samples were collected at more than one further time point: for patient 1, samples were collected at 9, 13, 14, 18, and 34 weeks; for patient 2, at 9 and 28 weeks.

### Histopathology

**FIGURE 3:**
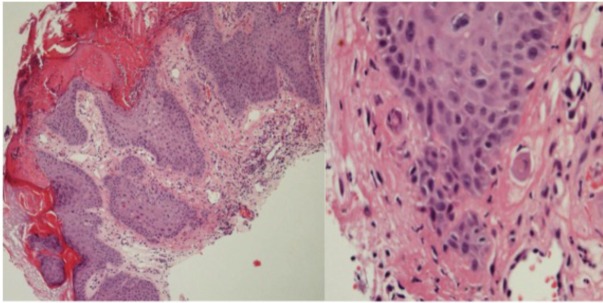
Histology of lesion V4: Micro invasion in an SCC from patient 1 with features of viral infection in overlying and adjacent epidermis.

Significant histological features of viral infection (i.e., koilocytosis and at least three of acanthosis, hypergranulosis, parakeratosis, hyperkeratosis or typical papillomatous architecture) were seen in all VW and AKs, 10/12 (83%) SCC, but no normal skin or VK samples (**Figure 3** and **Table 1**).

### Detection of Human Papillomavirus and Human Polyomaviruses

#### Human Papillomavirus Detection

Human papillomavirus DNA was detected in 18/19 (95%) VW, 9/12 (75%) SCC, 2/2 AK, 4/5 (80%) VK/SPs, and 3/6 (50%) normal skin samples (**Table 1**). HPV positivity was significantly higher in VW compared to normal skin (Fisher’s exact test: *p* = 0.0312,) but not in cSCC (Fisher’s exact test: *p* = 0.344). Multiple HPV types were detected in 28/37 (76%) of HPV positive samples, with a median number of 4 in VW and 3 in SCC compared with 0.5 in normal skin (two-sided Mann–Whitney *U*-test: *p* = 0.007 for VW and 0.13 for SCC).

Cutaneous beta-PV were detected in all HPV positive lesions, with HPV-8, 12, 24, 36, and 92 the most frequent types. Alpha genus HPV types (alpha-PV) were found VW only (4/19, 21%) and were mucocutaneous alpha-PVs (HPV28 and HPV57), but not low or high-risk mucosal alpha-PVs. One cSCC contained a gamma HPV type. Although the RHA detection methodology used for beta and alpha-PV detection is not strictly quantitative, band intensity provides a surrogate read-out for predicted viral load. Analysis of these data suggested that the majority of HPV positive samples were likely to be associated with low viral load: the bands identified were faint or very faint in all cases, with the exception of samples V10, V18, V20, V29, V44 in which strongly positive bands were obtained for HPV types 57, 12, and 80. Four of these five lesions were VWs.

In all patients except one, multiple lesions were analyzed. Clear patterns of HPV carriage for each individual emerged. For example, beta-PVs 8, 12, 24, 36, 92, 93, and 111 were detected in patient 1 across both benign and malignant lesions at different body sites; this patient’s normal, non-sun exposed skin sample also harbored HPV92 in addition to HPV5. Patient 2 had a spectrum of HPV types (HPV8, 12, 76, 80, and 57) that were concordant in VW at two separate body sites. Three other patients with more than one HPV positive lesion also had similar individual repertoires of HPV types across at least 2 lesions (patient 3, HPVs 22, 107; patient 4, HPV9; patient 7, HPV80). In the case of patient 6, all samples were negative, including two normal skin samples.

Only a minority of the 36 lesions with significant viral features on histological assessment had high levels of HPV DNA indicative of active infection. However, the 9 samples with no histological evidence of viral change (one cSCC, 2 VKs and 6 normal/perilesional skin samples) had significantly fewer HPV types detected compared to lesions with histological evidence of viral change (2-tailed Mann–Whitney *U*-test: *p* = 0.0114).

**FIGURE 4:**
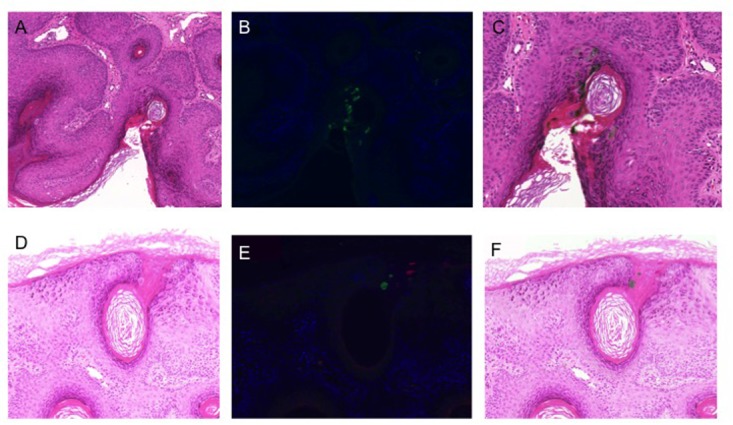
Human papillomavirus immunohistochemistry. **(A,D)** Hematoxylin and eosin stained sections of 2 different areas of lesion V10 (strongly PCR positive for beta-PV types 8, 12, 76, 80, and alpha-PV 57); **(B,E)** double staining immunofluorescence with antibodies to the E4 protein of HPV8 (green) and HPV2/57 (red); **(C,F)** H&E stained sections overlaid with the images from immunofluorescence microscopy to identify cells with productive HPV infection.

Immunohistochemistry was used to further investigate HPV DNA detection in a subset of 12 lesions. Although all had been HPV positive with multiple types detected, HPV protein expression was detected in only a single sample, a viral wart that had been strongly positive for both HPV57 and HPV80: both HPV57 and beta-PV were detected, but were expressed in spatially distinct cells within the lesion (**Figure 4**).

#### Human Polyomavirus Detection

Thirty lesional samples from 5 individuals were tested for the presence of HPyV (VW, *n* = 12; SCC, *n* = 11; VK/SP, *n* = 4; AK, *n* = 2; melanocytic, *n* = 1). Normal skin was not examined. The majority of samples were positive for at least one HPyV, albeit at low levels and independent of the presence or absence of significant histological viral features (**Table 1**). Individual HPyV positivity ranged from 0% positivity for TSPyV; 3.3% for HPyV9 (SCC, *n* = 1); 33% for HPyV6 (VW, *n* = 3, 25%; SCC, *n* = 4, 36%; VK, *n* = 3, 36%); 60% for HPyV7 (VW, *n* = 8, 67%; SCC, *n* = 6, 54%; VK, *n* = 3, 25%; AK, *n* = 1, 50%) to 73% positivity for MCPyV (VW, *n* = 9, 75%; SCC, *n* = 8, 73%; VK, *n* = 3, 75%; AK, *n* = 1, 50%; melanocytic, *n* = 1, 100%). For all viruses, the difference in positivity between BRAFi-cSCC and VW was not significant (Fisher’s exact test: *p* = 1 for HPyV9, HPyV6, MCPyV; *p* = 0.68 for HPyV7). Viral loads were generally less than one copy per thousand cells. The exceptions were four HPyV7-positive benign samples with viral loads ranging from 1 copy per 100 cells to one copy per seven cells and eight MCPyV-positive samples (two cSCC with viral loads of 2–5 copies per 100 cells and six benign lesions with viral loads from 1 copy per 100 cells to 1 copy per 4 cells).

#### Co-detection of HPV and HPyV

At least one HPV type and one HPyV type were co-detected in 22/30 (73%) of lesions. There was no significant correlation between specific HPV and HPyV types. However, it was noteworthy that lesions from patient 6, which were all negative for HPV, were also largely negative for HPyV, despite having significant histological features of viral infection. Similarly, the cSCC for patient 4 was negative for all beta-PV and HPyV types tested.

### Virus Status and Chromosomal Changes

**FIGURE 5:**
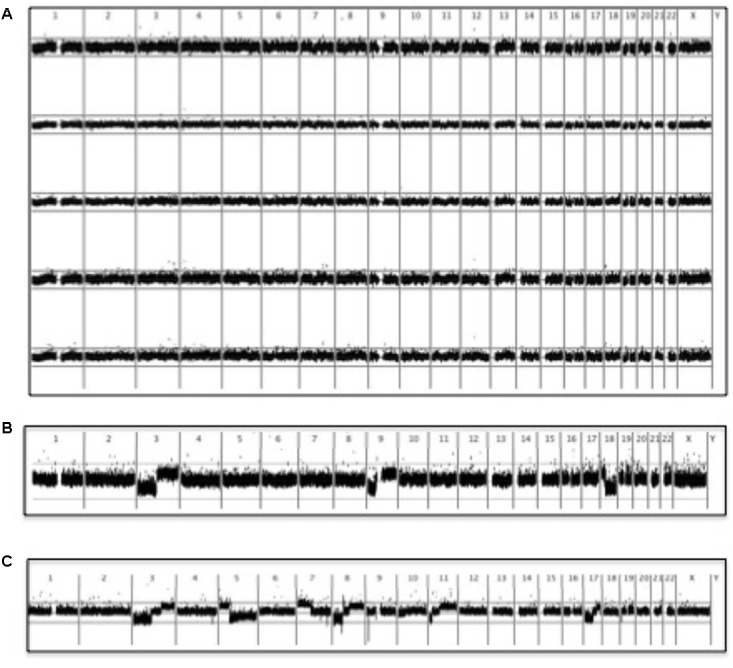
SNP microarray analysis of vemurafenib-associated cSCC. BRAFi-SCC did not display the gross chromosomal aberrations typically observed in sporadic cSCC (Purdie et al., 2009). A running average of 2 consecutive tumor: non-tumor signal value ratios is plotted on a log2 scale according to chromosomal position. Upper line represents log2(2) and lower represents log2(0.5). **(A)** Copy number profiles of 5 vemurafenib-associated cSCC **(B)** Comparison with copy number profile from a sporadic cSCC showing 3p and 9p loss: these changes were characteristic of well-differentiated cSCC (Purdie et al., 2009). Although chromosome 18 loss is also seen in this particular tumor, chromosome 18 aberrations were not as common as 3p and 9p changes in other sporadic well-differentiated cSCC (Purdie et al., 2009). **(C)** More extensive allelic imbalance in a sporadic moderately-differentiated cSCC (Purdie et al., 2009).

In order to examine gross chromosomal aberrations in vemurafenib-associated sSCC, we analyzed six well-differentiated cSCC from patient 1 using SNP array analysis to determine the signal values in tumor and paired non-tumor DNA at 250,000 SNPs throughout the genome. A comparison of tumor: non-tumor signal value ratios from vemurafenib-associated cSCC plotted according to chromosomal position with those from sporadic well-differentiated SCC analyzed in a previous study (Purdie et al., 2009) revealed that the patterns of gross chromosomal aberrations were significantly different: none of these 6 cSCC had gross chromosomal aberrations and there were significantly fewer chromosomal changes compared with sporadic well differentiated SCC (**Figure 5**). There were no clear correlations with virus status identified.

### Virus Status and Association With Specific Genetic Mutations

We have reported gene mutations identified by targeted sequencing in these 45 samples for *H, K* and *NRAS, NOTCH1 and 2, TP53, CDKN2A, CARD11, CREBBP, TGFBR1/2* and this has previously been presented in detail (South et al., 2014; Cammareri et al., 2016). In the current study we analyzed the association between these mutations and the presence of HPV and HPyV. HRAS mutations were identified in 5/12 (42%) SCC and 5/19 (31%) VW. Mutation did not correlate with sun-exposed sites or lesion type and no mutations were detected in normal skin samples (**Table 1**). There was no significant difference between HRAS mutated vs. HRAS wild type lesions and HPV or HPyV status. The same was true for virus status and mutations in each of the other genes examined. Although it was noteworthy that v35 – the most highly mutated SCC – was negative for all viruses tested, there was no evidence of a significant correlation between overall mutational burden and virus status.

### Virus Status and Clinical Response to Vemurafenib

There was no clear evidence of a significant association between virus status of lesions tested and prognosis in terms of disease progression and death from melanoma (data not shown).

## Discussion

We report HPV and HPyV analysis of 45 benign and malignant BRAFi-induced skin lesions from 7 individuals, including BRAFi-SCC and correlate this with clinical, histologic and genetic features. A high proportion of BRAFi-cSCCs had histologic viral wart-like features on histology, consistent with virus-driven processes, and the majority were positive for beta-PV, HPyV7 and MCPyV, which were co-detected in 73% of lesions tested. HPyV6 was found in one third of cases, but HPyV9 and TSPyV were rarely detected. As expected from previous studies of normal skin and hair follicles (Harwood et al., 2004; Bouwes Bavinck et al., 2010, 2017; Proby et al., 2011), normal skin samples also harbored beta-PV, but the HPV burden of individual types detected was significantly fewer than in lesional skin. Histologic evidence of virus infection appeared to correlate with HPV burden. However, viral loads were low in the majority of lesions and validation by immunohistochemistry for HPV was negative in all but one of 12 cases. Gross chromosomal changes characterized by SNP arrays in BRAFi-cSCC indicated that these tumors have significantly fewer chromosomal aberrations than non-BRAFi-cSCC, providing a further indication that additional cofactors may be involved. However, we were unable to establish clear correlations between the presence of either HPV or HPyV and specific genetic mutations or total mutational burden.

### Human Papillomaviruses and BRAFi-cSCC

Human papillomaviruses has been investigated as a potential viral carcinogen in BRAFi-cSCC since these drugs were first approved in 2011/2012: the rapid onset of skin lesions, their clinical morphology and viral wart-like histology all point to a possible role for HPV (Boussemart et al., 2013). Initial studies designed to detect alpha-PV infection by immunohistochemistry reported negative results (Anforth et al., 2012; Chu et al., 2012; Ko et al., 2013). A surrogate for alpha-PV infection in mucosal sites is p16 immunoreactivity and in two studies the majority of BRAFi-cSCC were found to strongly express p16 (Anforth et al., 2012; Boussemart et al., 2013), but this is not a consistent finding (Frouin et al., 2014). Both approaches are less sensitive than PCR-based detection methodologies that have been used in other studies, although the latter are often limited by the use of formalin-fixed paraffin-embedded tissue (FFPE) and/or PCR primers detecting a limited range of HPV types. Using an alpha-PV specific PCR/line probe assay method, Dika et al failed to detect alpha-PV in 9 FFPE VK samples (Dika et al., 2015). Using the same assay together with a degenerate PCR methodology (FAP59/64) capable of detecting cutaneous HPV types, HPV was not detected in 8 BRAFi-cSCC/KA FFPE samples (Frouin et al., 2014). Holderfield et al used the FPA 59/64 primers and additional degenerate primers (CP65/CP70 and CP66/CP69) and found 2/13 (15%) FFPE BRAFi-cSCC (Holderfield et al., 2014). Schrama et al. (2014) used E1 primers originally tested for alpha-PV types but theoretically capable of detecting any HPV type; in FFPE samples from 14 cSCC, 3 KA and one acanthoma, all were HPV positive, although the specific types detected were not reported. Subsequently, Cohen et al. (2015) used the most comprehensive degenerate PCR-based methodology in 69 FFPE BRAFi-cSCC and found all samples to be positive with predominantly beta-PV types of which HPV-17, HPV-38 and HPV-111 were the most common. Falchook et al. (2016) found 6/12 (50%) FFPE BRAFi-cSCCs to be positive, almost exclusively with beta-PV types including 12, 17, 24, 47, 124 and novel types but, as in our study, HPV positivity was not significantly different when compared to normal skin. However, we found a significant difference in HPV burden between lesional and normal skin in terms of the numbers of HPV types detected and it is possible that the total burden of HPV types as well as the specific types detected is relevant to potential pathogenic processes.

Against the background of these previous studies, our HPV detection methodology has notable strengths, although the data are still limited by small sample size. We have used a more comprehensive and sensitive approach to detecting HPV from alpha, beta, mu, nu, gamma genera with RHA and degenerate PCR/sequencing. We have also used fresh frozen tissue samples in order to reduce the likelihood of false negative results, which may occur with use of FFPE-derived DNA. Laser capture micro dissection allowed enrichment for lesional tissue and reduces the possibility of contamination by virus carriage in non-lesional tissue. Neither strategy for optimizing viral detection has been used in these previous studies. In addition, we used immunohistochemistry to try and understand the nature and significance of multiple HPV detection using a double staining technique not used in previous studies of BRAFi lesions.

Our study was not designed to address the functional significance of HPV detection in BRAFi-associated squamoproliferative skin lesions. To date, this has been attempted in only a few other studies. Ganzenmueller et al. (2013) used next-generation sequencing to look for viral transcripts indicative of active HPV infection: none were identified in 4 BRAFi-associated VK. Although this small study arguably calls into question a role for HPV, it is important to note that BRAFi-cSCC were not examined and the presence of very low abundance transcripts cannot be entirely excluded. In a compelling experimental approach, exposure to vemurafenib in a transgenic murine model (K14-HPV16 mice) of alpha-PV-driven cSCC was associated with an upregulation of the MAPK pathway and an increase in cSCC incidence from 22 to 70%. More than half of these tumors were *RAS* wild type, suggesting that vemurafenib and HPV may be cooperating to promote tumorigenesis in both the presence and absence of RAS mutations (Holderfield et al., 2014). Data from a more recent beta-PV transgenic mouse model provides evidence of significant synergism between beta-PV, UV, and BRAFi (Viarisio et al., 2017a). The K14-HPV38 E6/E7 transgenic mouse expresses the beta-PV HPV38 E6 andE7 oncogenes in the basal layer of the epidermis under the control of the cytokeratin K14 promoter and requires UV exposure for AK and cSCC development (Viarisio et al., 2011). Vemurafenib increased the number and size of UV-induced cSCC. All BRAFi-treated transgenic mice developed cSCC by 34 weeks in contrast to none of the wild-type mice and only one-third of untreated transgenic mice. Ras mutations were not detected, but MAPK upregulation was evident in HPV38 E6/E7 over-expressing keratinocytes (Viarisio et al., 2017a).

The mechanisms responsible for the synergism between HPV and BRAFi are speculative. However, experimental data indicate that upregulation of the MAPK pathway enhances alpha-PV replication, stability, and infectivity (Wang et al., 2009; Bowser et al., 2011). If this is also the case for other HPV types, then it is possible that BRAFi-induced paradoxical upregulation of MAPK in the keratinocytes of normal skin – which is likely to harbor beta-PV HPV (Harwood et al., 2004; Bouwes Bavinck et al., 2010, 2017; Proby et al., 2011) – also leads to enhanced replication and stability of these beta-PV. The resulting increased beta-PV viral load in normal keratinocytes may drive not only benign squamoproliferative lesions associated with BRAFi, but may also enhance synergism between the oncogenic effects of beta-PV and UV, independent of RAS status, which ultimately leads to increased carcinogenesis in the K14-HPV38-E6/7 transgenic murine model (Viarisio et al., 2017a). Consistent with this, experimental evidence increasingly points to a “hit and run” role for beta-PV in skin (Tommasino, 2017). In contrast to high-risk alpha-PV which are required for both initiation and maintenance of a malignant phenotype in mucosal carcinogenesis, the hit-and-run hypothesis proposes that beta-PVs are required only at an early stage of carcinogenesis, with beta-PV E6 and E7 oncogenes facilitating accumulation of UV-induced DNA mutations in the host genome by means of multiple mechanisms that, for example, target DNA repair and apoptosis leading to inactivation of cellular tumor suppressor proteins or activation of oncoproteins (Connolly et al., 2014; Howley and Pfister, 2015; Quint et al., 2015; Tommasino, 2017; Viarisio et al., 2017b). This is the “hit,” which ultimately leads to cellular transformation (Viarisio et al., 2017b). Viral oncogene expression is subsequently not required for maintenance of a malignant phenotype, rendering the viral genome dispensible and without consequence if lost from an established cancer – the “run.” This would explain the observations that beta-PV DNA loads are always significantly less than one viral genome copy per cell in cSCC and are generally higher in precancerous AK (Weissenborn et al., 2005), that viral transciptomes are absent from BRAFi and non-BRAFi-cSCC (Arron et al., 2011; Ganzenmueller et al., 2013), and are supported by together with findings in the recent murine K14-HPV38E6/7 transgenic models (Viarisio et al., 2018). However, although biologically plausible, this hypothesis remains to be definitively confirmed in human studies.

Such a hit-and-run role for beta-PV may also potentiate the deleterious effects of cSCC cofactors such as immune suppression and contribute to the higher susceptibility to cSCC of immune suppressed individuals such as solid organ transplant recipients (Harwood et al., 2017). We and others have previously shown that beta-PV carriage is significantly more common in the normal skin and hair follicles of immune suppressed individuals (Weissenborn et al., 2012) and, particularly in the presence of concordant beta-PV seropositivity, is associated with cSCC risk in both OTR (Proby et al., 2011; Bouwes Bavinck et al., 2017) and immunocompetent individuals (Bouwes Bavinck et al., 2010; Chahoud et al., 2016). Although detailed comparison of virus status in BRAFi- and non-BRAFi-associated cSCC might provide insight into the effects of BRAFi on the biological activity of beta-PV in skin, the small numbers of published studies in BRAFi-cSCC and the wide variations in HPV analyses used in these studies limit the power of such an analysis.

### Human Polyomaviruses and BRAFi-cSCC

We detected HPyV6 in one third of all 30 lesions tested, HPyV7 in 60% and MCPyV in 73%, with no significant differences seen between benign and malignant lesions. In most cases the loads for all viruses were low. In our series HPyV9 was negative in all cases and TSPyV positive in just one cSCC. Our data for MCPyV are very similar to the 72% positivity reported in a series of 18 FFPE BRAFi-cSCCs (Schrama et al., 2014) and 75% of 12 BRAFi-cSCC (Falchook et al., 2016). In contrast, Cohen et al detected MCPyV in only 22% of 58 FFPE benign and malignant FFPE lesions (Cohen et al., 2015) and a fourth study found MCPyV in only two of 19 VKs and none of 7 cSCCs (Frouin et al., 2014). Of the few studies to examine other HPyVs, Schrama et al. (2014) found HPyV6 and 7 in all FFPE samples (14 cSCC, 3 KA, and one acanthoma), generally at low levels, although HPyV6 was present at high level and detectable by IHC in one cSCC. In contrast, in FFPE samples of 19 VKs, 1KA and 7 cSCC, Frouin et al. (2014) found no HPyV6 positivity and HPyV7 in one VK only. Once again, in all previous studies samples were FFPE rather than frozen samples and this, together with the tumor enrichment and PCR approaches used in our study, may account for some of these differences in HPyV detection.

We did not test normal skin samples, but MCPyV, HPyV6 and 7 are well-established members of the normal skin virome, with HPyV9 and TSPyV significantly less common (Schowalter et al., 2010; Foulongne et al., 2012; Kazem et al., 2012; Wieland et al., 2014). MCPyV has previously been detected in 40–62% of skin swabs from normal individuals (Wieland et al., 2009; Schowalter et al., 2010) and HPyV6 and 7 have been detected in 14 and 11%, respectively (Schowalter et al., 2010). In comparison, HPyV9 and TSPyV were found in skin swabs from only 1/111 (0.9%) and 6/249 (2%) healthy individuals, respectively (Sauvage et al., 2011; Kazem et al., 2012). A previous study analyzed MCPyV in 9 FFPE normal skin biopsies from patients with BRAFi-associated proliferative skin lesions and found all to be negative, whereas 5/9 (56%) were positive for HPV of predominantly beta-PV types (Falchook et al., 2016). A case report by the same authors also failed to detect MCPyV in a normal skin of a patient with BRAFi-cSCC (Falchook et al., 2013). Other HPyV have not been analyzed in normal skin from BRAFi-exposed individuals. These data provide a possible signal that HPyV6 and 7 are overrepresented in BRAFi-cSCC, with our findings of 36 and 54% positivity, respectively. However, as normal skin is frequently positive for MCPyV, our findings in BRAFi-cSCC are less convincing. However, this interpretation is speculative and needs to be confirmed in future studies that specifically include matched normal skin samples from BRAFi-exposed individuals.

To date, there have been no functional studies specifically addressing the role of HPyVs in BRAFi-cSCC. The oncogenic potential of MCPyV is well established in MCC (Church and Nghiem, 2015; Paulson et al., 2017) and it is plausible that the large and small T-antigen oncoproteins may be relevant in BRAFi-cSCC. To date, none of the 12 other HPyVs have been implicated in causing cancer (Church and Nghiem, 2015). However, recent *in vitro* studies have indicated that MCPyV, HPyV6, and TSPyV can all induce cellular MAPK pathways (Wu et al., 2016, 2017a,b). It is therefore, plausible that such activity may act synergistically with BRAFi-induced MAPK upregulation and contribute to driving squamoproliferative lesions.

### Co-detection of HPV and HPyV

Human papillomavirus and HPyV were co-detected in the majority of virus positive lesions, usually at low copy number. There were no clear associations of specific types, although beta-PVs and MCPyV were most commonly co-detected, as previously reported (Falchook et al., 2013, 2016; Cohen et al., 2015). Our interpretation of these data is limited by the fact we have only examined normal skin for HPVs and not for HPyVs. However, co-detection of these potentially oncogenic viruses in BRAFi-cSCC remains an important observation. Although it is not possible from these data to conclude whether one virus type is biologically more relevant than another in either driving the virus features seen histologically, the potential for their interaction in playing an oncogenic role merits further functional investigation. In particular, it is plausible that the ability of HPyV to upregulate MAPK and the effects of MAPK upregulation on HPV replication, infectivity and stability may act synergistically in enhancing the oncogenic potential of both in contributing to the pathogenesis of BRAFi-associated squamoproliferative lesions.

### Virus Status and Genetic Alterations

We have previously used SNP array analysis to show that UV-associated well-differentiated cSCC have significantly different patterns of chromosomal aberrations compared with moderately and poorly differentiated cSCC (Purdie et al., 2009). Although BRAFi-cSCCs are histologically similar to well- differentiated sporadic cSCC, we have shown that they do not display the characteristic gross chromosomal aberrations typically associated with well-differentiated cSCC. This possibly reflects the shorter time course and more prominent role for MAPK upregulation induced by BRAFi, rather than through lifetime accumulated UVR-induced DNA damage, as seen in sporadic UV-associated cSCC (Lambert et al., 2014). Arguably, however, it also provides circumstantial evidence for alternative etiological agents such as oncogenic viruses.

At the individual gene level, as previously reported, we found a significantly higher level of HRAS mutations in BRAFi-cSCC compared with non-BRAFi-cSCC (South et al., 2014). In the current study, we specifically sought a possible association with virus status in HRAS mutated compared with HRAS wild type lesions. This may provide insights into the mechanisms underlying carcinogenesis in BRAFi-cSCC that are additional to mutant HRAS-associated upregulation of the MAPK pathway and, for example, related to viral oncogenes. However, no clear correlation emerged between virus and HRAS status. We also specifically looked for evidence of an inverse association between the detection of virus and mutational burden in terms of the numbers of mutated genes, as is seen in MCC (Harms et al., 2015; Wong et al., 2015; Goh et al., 2016; Becker et al., 2017; Starrett et al., 2017; Carter et al., 2018). Once again, no clear association emerged, although this study may have been underpowered to detect such an association.

## Conclusion

Despite the suggestive clinical and histologic evidence, a compelling experimental murine model and genetic evidence that HRAS mutations are absent in a significant proportion of BRAFi-induced squamoproliferative skin lesions, the contribution of HPVs and HPyVs to the development of these lesions suggested by results from previous studies remains inconclusive. The data presented here provide further circumstantial evidence for a possible role for HPV and HPyV. They also point to possible synergistic interactions between these potentially oncogenic skin viruses. Given the major increase predicted in adjuvant use of these agents in the near future, further research into the role of these and possibly other existing or novel members of the human skin virome is justified and may provide insights into the pathogenesis of other BRAF-induced skin disorders and malignancies.

## Author Contributions

CH, KP, CP, and IL conceived and designed the study. CH, KP, HR, EM, MF, JD, HG, AS, GI, IL, and CP developed the methodology. CH, KP, HR, EM, MF, JD, HG, MS, GI, and AS acquired the data. CH, KP, HR, EM, MF, JD, HG, MS, AS, GI, and CP analyzed and interpreted the data. CH, KP, JD, MF, HR, IL, and CP wrote, reviewed, and revised the manuscript. CH, CP, and IL supervised the study.

## Conflict of Interest Statement

The authors declare that the research was conducted in the absence of any commercial or financial relationships that could be construed as a potential conflict of interest. The handling Editor declared a past co-authorship with several of the authors MF, CH, and CP.
